# Preparing historically underrepresented trainees for biomedical cancer research careers at Huntsman Cancer Institute/University of Utah Health

**DOI:** 10.1080/10872981.2021.1929045

**Published:** 2021-05-23

**Authors:** Ana María López, José E Rodríguez, Kathryn Browning Hawes, Anna Marsden, Don Ayer, Donna Harp Ziegenfuss, Kola Okuyemi

**Affiliations:** a Professor and Vice Chair of Medical Oncology, Sidney Kimmel Medical College at Thomas Jefferson University, Philadephia, PA, USA; b Professor of Family Medicine and Associate Vice President for Health Equity Diversity and Inclusion, University of Utah Health, Salt Lake City, UT, USA; c Office of Health Equity, Diversity and Inclusion, University of Utah Health, Salt Lake City, UT, USA; d Huntsman Cancer Institute, University of Utah, Salt Lake City, UT, USA; e Marriott Library, University of Utah, Salt Lake City, UT, USA; f Professor and Chair, Department of Family and Preventive Medicine, University of Utah Health, Salt Lake City, UT, USA; g Professor and Department Chair of Family & Preventive Medicine

**Keywords:** Pipeline program, health equity, partnership, health care career, underrepresented trainees

## Abstract

Given the well-documented inequities in health care outcomes by race, ethnicity, and gender, many health career pipeline programs have focused on supporting the development of a diverse and inclusive workforce. The State of Utah, is vast, but sparsely populated outside the Salt Lake City metropolitan area. More than 96% of our nearly 85,000 square miles is designated rural (<100 people/square mile) or frontier (<7 people/square mile). The Salt Lake City area is home to the Hunsman Cancer Institute, the only NCI-designated Comprehensive Cancer Center in the region, also noted the limited diversity in the biomedical cancer research workforce. Our primary objective was to increase the number of underrepresented trainees who pursue higher education with the goal of a career in cancer research. *PathMaker* is a regional, competitive pipeline program that nurtures high school or undergraduate trainees from historically underrepresented backgrounds towards a career in cancer research. Our faculty and staff team collaboratively developed a cohort model curriculum that increased student awareness of research career options; provided academic and professional development, cultural and social support, evolutionary success strategies, active mentorship, and leadership skill development; and fostered an environment of continuous evaluation and improvement. Since pilot program initiation in May 2016, the *PathMaker Research Program* (*PathMaker*) has engaged a total of 44 underrepresented trainees in cancer research labs at Huntsman Cancer Institute, the majority still in college. Eleven trainees graduated college: five employed in STEM, one pursuing a PhD in STEM; two in medical school, and three are lost to follow-up. Alumni report high levels of satisfaction with *PathMaker* and will be followed and supported for academic success. *PathMaker* is a replicable model to increase diversity and inclusion in the biomedical cancer research workforce.

## Introduction

The drop in historically underrepresented students (HUS) on the path towards becoming independent scientists has been well-documented [1,2]. In 2010, Latinx, Black, and American Indian persons represented more than a third of the population in the USA [3]. Doctorate degree recipients generally serve as the entry point for independent investigators into the biomedical research workforce. In 2010, the number of PhD recipients in science, technology, engineering, and mathematics (STEM) fields to persons who identify as Latinx and Black was approximately 7% [4]. (The authors acknowledge the use of varying terms, i.e., Hispanic, Latino/a, or Latinx. Our cohort students and trainees with this identity expressed a preference for the term Latinx; therefore, Latinx is used throughout the manuscript).

The Mountain West region, the catchment area for Huntsman Cancer Institute (HCI), encompasses the five-state area of Utah, Wyoming, Montana, Idaho, and Nevada and includes federally recognized American Indian Nations, rural and frontier populations, and an increasingly diverse population[5]. For example, Utah is home to a growing Latinx population (approximating 450,000, 14.2%), part of the Navajo Nation (the largest Indian reservation in the USA), and the highest percentage of Pacific Islanders in any state outside of Hawaii[6]. More than 60,000 refugees reside in Utah[7]. With the addition of other immigrant populations, over 120 languages are spoken in the state[8].

Programs to enhance the diversity of the clinical health care and research workforce [9–14] may begin as early as middle school and include academic and summer research opportunities. These summer academic and research skill development experiences [15] have been associated with increased STEM PhD program enrollment. For greatest impact, career opportunity awareness sessions, mentorship experiences, time for reflection, and leadership development [16,17] experiences can supplement and support the educational experience.

The *PathMaker Research Program (PathMaker)* seeks to increase the number HUS pursuing biomedical cancer research. It was our hypothesis that a residential research experience in a supportive environment that included co-curricular experiences and ongoing engagement and support would be most likely to yield success. Co-curricular experiences included cultural and social support, mentorship, and leadership skill development. Our collaboratively developed multi-faceted cohort curriculum (Please see Table 1) supports trainees during the transition from high school to college through specific outcomes. *PathMaker* is funded by a Supplement to the Cancer Center Support Grant (3P30CA042014-27S6) and receives the highest level of support from HCI. *PathMaker* leadership includes a senior HCI faculty leader as Program Director, a Program Coordinator, and a broad-based advisory committee that supports Cancer Training and Career Enhancement programs at HCI. The success of the *PathMaker* experience has resulted in a successful R25 application (1R25CA240171-01).

**Table 1.** *PathMaker* curricular components

Goal	Method	Outcomes
*Increase awareness*, knowledge, and interest in pursuing education towards a career in biomed ca research	ResearchExperiences: Basic sciences Clinical sciences Population sciences	Program satisfaction High school completion Undergraduate acceptance Undergraduate retention and completion Academic accomplishments Pursuit of a biomedical cancer research career
*Increase the capacity* of trainees underrepresented in biomedical cancer research	Developmental Experiences: Academic and professional success Cultural and social support Evolutionary success strategies Active mentorship Leadership skill development	

This paper outlines the program components, presents five-year outcomes and lessons learned, and addresses future considerations to support the success of HUS entering careers in biomedical cancer research.

## Methods

### Program description

*PathMaker* is a residential summer program based at HCI, University of Utah Health (UUH). Initially funded as a one-year pilot program through the Office of Health Equity and Inclusion (UUH), extramural competitive funding was successfully achieved for the subsequent years through the National Cancer Institute’s Continuing Umbrella of Research Experiences (CURE) Supplement. *PathMaker* focuses on the transition between high school and college and provides incoming HUS (high school seniors and undergraduates) with an enriching cancer research experience; academic, professional, cultural, and social support; mentorship; and leadership skill development. Social support includes extensive peer networking and family engagement.

**Recruitment**: *PathMaker* uses email, web, social media (Facebook and Twitter), and engagement with diversity-focused organizations to recruit trainees from high schools and colleges in the Mountain West. Word of mouth referrals by prior participants and community partners has also contributed to successful recruitment. Regional recruitment includes outreach through an existing extramurally funded regional partnership, the Geographic Management of Cancer Health Disparities Program (GMaP, 3P30CA042014-26S3). *PathMaker* also partners with diversity focused organizations that support HUS’s success in higher education. These included regional university organizations: TRiO and Upward Bound Programs, Latinos in Action, and the Black Student Union; university chapters of the Society for the Advancement of Chicanos and American Indians in Science (SACNAS); and the Utah State Board of Education Title VI: American Indian Education Program. High school outreach engaged high school advisors, educators, and every high school principal in the state.

**Application and selection process**: Applicants must have completed their junior year of high school prior to participating in the program and must demonstrate an interest in health sciences. Completed applications include demographic factors: race/ethnicity, self-identified gender, age, current school, home zip code, and parents’ educational attainment. Applicants submit a 1–2 page personal statement, copy of their most recent transcript, and two letters of recommendation. Letters of recommendations may be from an employer, tribal leader, or mentor, and from a teacher. A copy of the most recent tax return is required if the student seeks consideration based on economic disadvantage. All application materials are submitted electronically to the *PathMaker* Program Coordinator. Incomplete applications are not reviewed.

All applications are reviewed by the *PathMaker* Selection Committee. The *PathMaker* Selection Committee is diverse and inclusive of Black, Latinx, American Indian, Pacific Islander, LGBTQIA+, Asian, and non-Latinx, white members and represent multiple stakeholders across UUH. Members review and score each application using a standard scoring sheet. Review criteria include academic accomplishments; HUS identity; economic, social, or educational disadvantage; personal statement; and letters of recommendation. After all scores are entered, compiled and averaged, the selection committee meets to discuss and review the top-ranking applicants. In general, eight trainees are selected with three alternates.

**Student Peer Mentors**: The Selection Committee also reviews applications for Student Peer Mentors (SPMs). Two SPMs are hired each year. The SPMs live with the *PathMaker* trainees in residential housing on-campus. As SPMs they serve as residential advisors, facilitators, and overall guides to the undergraduate experience. SPMs are generally senior level undergraduate students at the UU who have previously lived on campus as a residential advisor. SPMs complete the UU Youth Protection Training course prior to the *PathMaker* trainees’ arrival on campus. The SPMs work closely with the *PathMaker* Program Coordinator to manage all day-to-day aspects of the experience. They attend all educational activities with the cohort.

**Mentors**: *PathMaker* mentors are HCI investigators whose research focuses on cancer aspects related to the basic, clinical, or population sciences. HCI principal investigators (PIs) are invited to participate as a mentor for *PathMaker*. Mentors are invited to a Mentor Orientation prior to the start of the *PathMaker* residential experience. Throughout the program, there is close communication between the Program Director, the Program Coordinator, the Student Peer Mentor, and the mentor. Formal, face-to-face student evaluation takes place at week 4 and upon program completion. In total, 27 HCI investigators have participated in *PathMaker*: 11 (40%) have participated with more than one cohort of trainees; 16 (59%) female, 11 (41%) male; 20 white, non-Latinx (74%), 3 Latinx (11%), and 4 Asian (15%).

**The Summer Experience**: Upon enrollment, trainees receive a UU identification card which welcomes them and facilitates access to UU resources. The trainees, SPMs, and Program Coordinator communicate regularly using a mobile application called GroupMe, which allows for real-time group or individual conversations.

Upon acceptance, trainees receive a booklet of the HCI research labs with information about the participating research teams. All trainees and their families are invited to a welcome reception held the week prior to the *PathMaker* residential start date. At that time, the trainees meet the *PathMaker* Program team and participating mentoring research labs. After the welcome reception, trainees submit their top three lab choices to the *PathMaker* Program Coordinator.

The lab-intensive training course orients the *PathMaker* trainee to lab safety, basic lab techniques, and population research tools that they are likely to encounter during their research experience. The training is experiential in nature. All trainees complete HIPAA and CITI training. Additional trainings may also be offered depending on the work in the lab.

Trainees participate in weekly educational and social activities which are intended to bring the cohort together, expose them to various career paths and resources, and develop their professional skills and resiliency (see Table 2).

**Table 2.** *PathMaker* program activities

Week 1–2	Week 3	Week 4–12		
InitialEngagement	Preparing for Research Exp	Research Experience	Developmental Experiences	
			Increase Awareness	Increase Capacity
Pre-arrival Preparation-Virtual: f HIPAA Training CITI Training	Welcome Reception Research Placement Orientation Lab Safety Intensive Training Pop Sci 101 Training	Individual Mentored Research Experience with Weekly Lab Meetings and Mentor Meetings Cancer Prevention and Screening Education Poster Preparation Summer Research Symposium Closing Honoring Ceremony and Reception: for Trainees, Families, Mentors	Graduate School Expo Biomedical Research Career Seminar Health Science Graduate Student Panel Health Science Professional Panel Financial Skills and Support Financial Literacy Competency Medical Simulation Lab Tour College 101 Workshop Professional Development Expo: breakout sessions included, *How to Write a Personal Statement, Boosting your Resume/CV, How to Fund Your Education, Impostor Syndrome: Creating Strong Identities*, and *Interviewing Skills*.	How to Read a Research Article Writing an Abstract Literature Reviews Creating Effective Research Posters, Navigating Discrimination Bystander Education Diversity Workshop Mindfulness Growth Mindset Mental Health Awareness Reflection as Part of Research Career Skills Mentoring Workshops ProfessionalCommunication Mentorship Panel: Meet the PI

*PathMaker* is well integrated with other residential summer programs, e.g., Native American Research Internship (NARI), the Genomics Summer Research Internship for Minorities (GSRM), and Summer Program for Undergraduate Research (SPUR). Both NARI and GSRM focus on providing research experiences for trainees traditionally underepresesented in biomedical research. *PathMaker*, NARI, GSRM and SPUR collaborate on weekly events in order to build a larger community for the summer research trainees.

The *PathMaker* cohort meets weekly to reflect, debrief, and share successes and setbacks. These sessions are facilitated by the SPMs who offer reflective and motivational listening, share experiences, and offer the trainees support and guidance.

The summer experience concludes with *PathMaker* trainees presenting their research posters at the annual Summer Symposium for Undergraduate Research sponsored by the UU Office of Undergraduate Research. The Symposium provides the trainees with the opportunity to engage with all UU PIs, trainees, and trainees engaged in summer research experiences and generally hosts more than 100 posters. The poster presentations are followed by an Honoring Ceremony for trainees, families, and friends. The ceremony includes a ‘graduation’ with certificate presentation, reception, group photo, and delivery of a digital Yearbook that includes a record of their experiences along with an individual research page with their poster.

***Impact Assessment****: PathMaker* outcomes include program satisfaction; undergraduate retention and completion; academic accomplishments including scholarship awards; and progress on the path to pursue a biomedical cancer research career. For high school trainees, additional outcomes include high school completion and undergraduate acceptance. Our program outcomes assessment was reviewed by the UU Institutional Review Board and was deemed to be exempt.

Participants completed ongoing concurrent programmatic assessments during the experience. They also received a follow-up survey. This survey asked a single question: How has *PathMaker* changed or shaped your career or educational path. NVivo software was used to code open-ended responses. An inductive thematic analysis process of open coding, axial coding, and selective coding was used to identify themes in the responses[18]. Emerging themes were verified.

All *PathMaker* data are entered into a de-identified database. *PathMaker* staff maintain ongoing engagement with the trainees through email correspondence, social media (Facebook, Twitter, LinkedIn), and monthly newsletters. This post-experience engagement allows for ongoing support, opportunities for future work (i.e., Diversity Supplement applications), reunion experiences, and informal mentorship opportunities across cohorts. These interactions maintain current contact information; educational progress, recommendation requests; accomplishments and honors; and current research activities. These outcomes may be correlated with demographics; cohort year and program dates; mentor name (grant title and number); high school; and college. University acceptance status – including multiple acceptances, scholarship award data, and post-graduation activities were obtained by a post-summer experience completion survey through email, LinkedIn profile, or follow-up text to individual *PathMaker* participants. All trainees set up an eRA Commons ID and are added to the NIH CareerTrac System where trainee outcomes are supported throughout their career.

## Results

**Matriculation**: *PathMaker* admission is competitive. Table 3 outlines matriculant demographics and acceptance rates. Consistent with national classifications[19], underrepresented populations in biomedical cancer research were identified as Black (B), American Indian/Alaskan Native (AI/AN), Pacific Islander (PI), and Latinx (L). Women (W) were considered underrepresented since in Utah, women comprise 23.5% of the STEM workforce compared to 28.8% in the US [20] and make up 26.4% of the physician workforce compared to 35.7% nationally[21]. Similarly, although Asian trainees or trainees of Asian descent are often not considered underrepresented, in Utah, the Asian population is 2.4% compared to 5.5% nationally. Asian matriculants presented with intersectional HUS identities: women (69%), first-generation college trainees (85%), low socioeconomic status (46%), and refugee backgrounds (62%). Refugee, socioeconomic, and first-generation college student status, LGBTQ+, rural or frontier dwellers, and disability were also considered as factors representative of underrepresentation in the biomedical cancer research workforce.

**Table 3.** Matriculants

	Summer 2016		Spring 2017		Summer 2017		Summer 2018		Summer 2019		Total	
	***N***	**%**	***N***	**%**	***N***	**%**	***N***	**%**	***N***	**%**	***N***	**%**
AA or Black	2	25	1	20	0	0	2	15	2	20	7	16
AI/AN	0	0	1	20	0	0	1	8	0	0	2	5
Asian	3	38	0	0	4	57	3	23	3	30	13	30
White, Non-Latinx	1	13	0	0	0	0	1	8	0	0	2	5
Latinx	2	25	3	60	3	43	6	46	5	50	19	44
Female	6	75	3	60	5	71	10	77	5	50	29	67
Male	2	25	2	40	2	29	3	23	5	50	14	33
Gender Non-conforming	0	0	0	0	0	0	0	0	0	0	0	0
First Generation	5	63	5	100	6	86	10	76	8	80	34	79
Low SES	4	50	2	40	2	29	4	31	4	40	16	37
Rural	1	13	2	40	1	14	3	23	1	10	8	19
Refugee	3	30	0	0	2	20	1	10	4	40	10	23
Disability	0	0	0	0	0	0	1	8	0	0	1	2
Average High School GPA^a^	3.67		3.44^b^		3.58		3.73		3.72		3.63	
High	3.96		3.96		4.00		4.00		3.94		3.97	
Low	3.30		3.00		2.80		3.20		3.36		3.13	
High School Senior	5		0		4		8		5		22	
Freshman, Undergraduate	3		2		2		4		3		14	
Sophomore, Undergraduate	0		1		1		1		1		4	
Junior, Undergraduate	0		1		0		0		0		1	
Senior, Undergraduate	0		2		0		0		0		2	
Matriculants by Cohort	8		5		7		13		10		43	
Total # of Applicants	17		11		32		29		79		168	
Acceptance Rate	47%		45%		22%		45%		13%		26%	

^a^Average HS GPA: admitted students UU: 3.5 [26].

^b^College GPA: all entrants were college students.

Most trainees enrolled at the UU (31 of 43). *PathMaker* trainees are more diverse than the overall UU student population (Table 4): Black (+15%), Latinx (+29%), AI/AN (+6%), Asian (+26%), and female (+22%) representation. Seventy-seven percent of alumni have declared a STEM major (Table 5).

**Table 4.** Demographics: University of Utah trainees and *PathMaker* trainees at the UU

	U OF U		PathMaker Trainees		% Increase
	N	%	N	%	
Asian	2559	6	10	32	533
Black or African American	506	1	5	16	1600
White, Non-Latinx	29,319	72	2	6	−92
International	1653	4	NA	NA	NA
Latinx	4130	10	12	39	390
Multiple Race/Ethnicity	1436	4	0	0	NA
American Indian/Alaska Native	197	0	2	6	600
Pacific Islander	246	1	0	0	−100
Unknown	734	2	0	0	NA
Female	20,113	49	22	71	144
Male	20,667	51	9	29	−43
**Total**	**40,780**		**31**

**Table 5.** Undergraduate field of study for *PathMaker* alumni

Major	Overall		U U	
	*N*	% (*n* = 43)	*N*	% (*n* = 33)
Athletic Therapy	1	2	0	0
Biology	13	30	11	26
Honors Biology	2	5	2	5
BS/MD Biochem/Molecular Biology	1	2	0	0
Chemical Engineering	1	2	1	2
Chemistry	1	2	1	2
Computer Science	2	5	1	2
Kinesiology	3	7	3	7
Mechanical Engineering	1	2	1	2
Neuroscience	1	2	0	0
Nursing	1	2	1	2
Physics	1	2	0	0
Pre-Med	3	7	3	7
Psychology	1	2	0	0
Recreational Therapy	1	2	1	2
Undecided, Undeclared, or Unknown	4	9	2	9
Non-STEM-related majors	6	14	6	14
**Total**	**43**		**33**	

**Program Perceptions**: *PathMaker* participants stated that program was beneficial in many ways. The matching of trainees and mentors has proven successful. In addition to positive comments about the logistics and value of the *PathMaker* program, five themes were identified (Table 6). As an example, in discussing how *PathMaker* ‘Opened their Eyes to Research’, trainees spoke about how windows and doors of opportunity were opened to them. One student commented, ‘My experience there introduced to me scientific research and opened up many doors for me including finding future research opportunities.’

**Table 6.** Themes identified from the qualitative analysis

Theme	Coding Frequency (% of the data)
Opening Eyes to Research	25.9
Exploring Interests to Find Direction	23.3
Discovering Career Opportunities	19.8
Building Confidence and Character	19.0
Making Connections	12.1

**Academic success**: *PathMaker* trainees have secured NCI research funding and participated in prestigious undergraduate research programs. One of the spring 2017 trainees and PI/mentor teams was awarded an NCI Diversity Supplement in 2018. This Award, which supports a supplementary project to the existing funded award, funds the trainee’s work for at least two years. All trainees and PI/mentor teams are encouraged to consider Diversity Supplements to continue to fund their collaboration.

*PathMaker* trainees have participated in the Harvard Research Experience for Undergraduates, John Hopkins School of Medicine Summer Internship Program, and the Broad Summer Research Program (BSRP) though Harvard and MIT. The BSRP is a highly competitive research program designed for trainees to pursue genomics-based research. After graduating, one trainee accepted a full-time research positon at the Broad Institute. Trainees have successfully obtained scholarships and have demonstrated that they are competitive applicants to nationally recognized colleges.

Of the 43 alumni (1 trainee left the program within days of starting and has been lost to follow-up), 32 are enrolled an undergraduate program: 1 community college, 23 UU, 8 other undergraduate program (3 in programs ranked in the top 15 in the US). Eleven have completed their undergraduate degrees: five are employed in STEM; one is pursuing a PhD in STEM; two are in medical school, and three have been lost to follow-up.

## Discussion

*PathMaker* trainees are contributing to the diversity of the biomedical workforce as they uniformly identify as HUS in the biomedical sciences as outlined above. All accepted incoming high school seniors have completed high school and are enrolled in college. Of those who have declared a major, 77% are pursuing a STEM major. Of those who have graduated college, all, with known current status, are engaged in a career in science or are pursuing ongoing education towards a career in science.

Qualitative data support our hypothesis that embedding the *PathMaker* research experience within a supportive environment, one with the opportunity to build confidence, connect with mentors, and identify the intersection of interests with career opportunities, may bolster outcomes. The value of mentoring and an authentic research experience is a common theme in the undergraduate research literature[22]. Findings also indicate that an early research experience builds confidence and character and impacts general college success[23].

Our data demonstrate the theme of *Making Connections* as a thread that spanned all themes. Linn et al. (2015) contend that STEM trainees need to have an authentic research experience but also experience the context of research and practice[24]. Mentoring, communicating with researchers about research, and learning about research dissemination were all cited as value-added aspects the experience. *PathMaker* trainees had a research experience within a rich contextual environment that facilitated their ability to make explicit the connections between their new knowledge, their research experience, and their future career aspirations. *PathMaker* is an example of a high impact intervention that engages trainees in the act of learning and helps them make connections[25].

*PathMaker’s* success has fueled the development of new opportunities to support the development of HUS in biomedical cancer research. HCI launched a two-week summer research program in Summer 2019 for rural high school trainees who had not completed their junior year. This residential program allowed the partnering of a younger student with a *PathMaker* trainee for a peer mentoring experience. The experience took place during the last two weeks of the PathMaker program. The rural high school student shadowed their partner *PathMaker* trainee in the research lab, attended the educational sessions, and supported the *PathMaker* trainee at the final poster symposium. We anticipate that this program will create a natural entry point for the rural trainees to enter the *PathMaker* program and expect several applications for the subsequent summer *PathMaker* program.

Our experience with *PathMaker* served as the cornerstone for our successful *PathMaker Programs for Cancer Research* application funded through the National Cancer Institute (NCI) Youth Enjoy Science Research Education Program (YES R25) (1R25CA240171-01) to HCI in September 2019. This award will fund 14 *PathMaker Scholar* trainees for two consecutive summers, a teacher education component, *PathMaker Bridge*, and a middle school educational outreach component, *PathMaker Connect*.

## Conclusion

*PathMaker* has served as a formative experience for its alumni. We do not contend that *PathMaker* is *the* one experience that is singularly responsible for a student’s success nor that there is only road to success. It is not our goal to demonstrate causality. We consider success to be the result of multiple, and likely, a matrix of factors. Our goal is to provide students interested in science with experiences within a supportive environment where they can continue to grow and thrive towards their stated goal. *PathMaker* is early in its history. It has demonstrated success. We share our approach as it is replicable and can be tailored to your own community. Our trainees have found it valuable, and it has served as a foundation for additional funded interventions to support the diversity of the biomedical research workforce. We will continue to follow our cohorts. We will continue to support our mentees, and we look forward to learning and sharing their ongoing path making success in future reports.
